# A University Appeal

**Published:** 1920-04-24

**Authors:** 


### A UNIVERSITY APPEAL.

Already the Press has referred in laudable terms to the fact that the University of Liverpool, in taking the initiative in the matter of making a public appeal, has dealt a death-blow to the traditional idea that a University cannot avail itself of the recognised method of attracting public attention to its needs and work. On a later page (p. 89) we publish an article which should go far towards bringing home a realisation of the paramount national importance of encouraging advanced and specialised education—be it medical or otherwise—and particularly of providing adequate accommodation, efficient laboratories, and a teaching staff drawn from the ablest men it is possible to obtain. At the moment there is a great wave of enthusiasm among those people whose eyes have been opened by the events of the past few years. Not only have they learnt that victory is not to the soldier alone, but also that the educated, versatile mind is the most urgent personal and national necessity of the day. Unless full advantage can be taken of this progressive tendency among the youth of the country, or if chance obstructions arise, then there is grave danger that gradually the lethargic conditions of 1914 may again develop throughout the land and the happenings of that fateful time be repeated—but with, perhaps, an ending far from satisfactory to Great Britain and her interests.

One other point we would bring to notice—namely, that the preliminary tests which these candidates for higher education must pass should be made sufficiently exacting to ensure that only those fully suited to the difficult paths of higher scientific life should attempt to progress along them. This selective process is that which a great university typically provides, and, once this requirement has been fulfilled, the rest should become easy, for on the selected or super-minds depends the Empire's strength."

In Liverpool the present pressure is so great that additional facilities are a matter of urgent necessity, as the number of undergraduates has increased from 1,522 in 1919 to 2,455 in 1920, and many are being turned away in spite of the fact that wooden huts have been erected in the quadrangle and grounds till there is no room for more, open courts have been roofed over, and many private dwelling-houses pressed into service for the more important departments. As the issued memorandum explains, money is therefore urgently needed, mainly for the erection of new buildings. In the past the University has depended for assistance in this respect upon the generosity of a handful of benevolent persons with the interests of Education at heart. Unfortunately, the present needs are so great that they involve a far larger sum than can be expected from so limited a circle.

Of the University itself, we would draw attention to the fact that its doors are open, without distinction of sex, class, or geographical origin, at moderate fees to all young men and women who prove their capacity for higher education by passing its matriculation examination. The facilities which have been provided deserve every possible recognition, and it has now become imperative to impress upon the public attention the supreme importance to the nation of the prompt removal of any countervailing influences. The demand must be met for an education of the highest possible standard for the rising generations, not only in Liverpool, but throughout the country. The present appeal, however, comes as one of particular urgency when emanating from a great national centre and port in which it is absolutely necessary that a powerful first-class educational element should be maintained. Liverpool has always provided first-class teaching in Medicine, Law, Science, Commerce and the Arts, and in some sections can claim to stand pre-eminent. The departments of Tropical Medicine and Oceanography, to take two examples, are the most advanced in the world, as indeed they should be in a great port where thousands of men go out daily to meet the dangers of the tropics and the seas. In short, a most deserving appeal is made to all classes, and an invitation to pay a personal visit to the University is cordially extended to those who sympathise with the difficulties of this educational emergency. We trust that the Appeal Director, at 4 Moorfields, Liverpool, to whom all communications should be addressed, will later be able to give us an account of complete and unqualified success.

